# Mitochondrial Quality Control and Pathogenic Signaling Networks in Parkinson’s Disease

**DOI:** 10.3390/cimb48070645

**Published:** 2026-06-23

**Authors:** Xiaobing Zhang, Huiyu Li, Jiaxin Zhao, Jiawen Tang, Xiaoqing Li, Pengjing Li, Qingyun Zhao, Qi Wang, Wei Zou

**Affiliations:** 1 School of Public Health, Kunming Medical University, Kunming 650500, Chinazhaoqingyun@kmmu.edu.cn (Q.Z.); 2 Experiment Center for Medicial Science Research, Kunming Medical University, Kunming 650500, China

**Keywords:** Parkinson’s disease, mitochondrial malfunction, oxidative stress, neuroinflammation, m^6^A modification, PINK1/Parkin pathway, therapeutic target

## Abstract

The second most prevalent neurodegenerative illness in the world, Parkinson’s disease (PD), currently has no viable treatments. Although it is yet unknown if mitochondrial dysfunction is an initial event or evolves as a result of neurodegeneration, it is thought to be a crucial component of Parkinson’s disease etiology. From the perspective of mitochondrial quality control (MQC), which includes PINK1/Parkin-mediated mitophagy, mitochondrial dynamics, and mitochondrial proteostasis, this article examines mitochondrial dysfunction. Together, these processes preserve mitochondrial homeostasis and prevent the buildup of damaged mitochondria. Dysfunctional mitochondria gradually build up and cause oxidative stress and aberrant cellular signaling when mitochondrial quality control is compromised. According to available data, mitochondrial reactive oxygen species (mtROS) primarily worsen pre-existing mitochondrial damage by encouraging α-synuclein aggregation, cardiolipin remodeling, and dopamine oxidation. In addition, innate immune pathways like cGAS–STING and TLR9 signaling can be triggered by mitochondrial damage-associated molecular patterns (mtDAMPs), especially mitochondrial DNA, which can lead to long-term neuroinflammatory reactions in PD. While new research suggests that m^6^A RNA modification may be involved in the regulation of mitochondrial stress, the PINK1/Parkin pathway is crucial for maintaining mitochondrial homeostasis. Therapeutic approaches that target mitophagy augmentation, neuroinflammatory signaling, and mitochondrial protection have garnered increasing attention. In an attempt to improve mitochondrial function and lessen persistent neuroinflammatory activation, future research will probably need to concentrate on combination treatment techniques.

## 1. Introduction

Parkinson’s disease (PD) is the second most common neurodegenerative disease worldwide and represents a major health and socioeconomic challenge, especially in older populations [1]. Some of the clinical features of PD that gradually impair motor function and quality of life include resting tremors, rigidity, and postural instability. In addition to these motor manifestations, patients frequently experience a range of non-motor symptoms, including hyposmia, sleep issues, autonomic dysfunction, cognitive decline, and psychiatric disorders. It is important to keep in mind that a lot of these non-motor symptoms might appear in the prodromal stage before typical motor symptoms [2].

Neuropathologically, PD is characterized by progressive dopaminergic neuronal loss in the substantia nigra pars compacta and by the presence of Lewy bodies containing aggregated α-synuclein [3]. Loss of nigrostriatal dopaminergic neurons disrupts dopamine signaling and basal ganglia circuitry, ultimately leading to the characteristic motor abnormalities observed in PD [4]. Nevertheless, the molecular basis underlying the selective vulnerability of dopaminergic neurons has not yet been fully clarified.

Oxidative stress, protein aggregation, mitochondrial dysfunction, and neuroinflammation are considered major contributors to Parkinson’s disease pathogenesis [5]. A key and unifying feature in the pathophysiology of PD and other age-related neurodegenerative illnesses, such as Alzheimer’s disease, is mitochondrial dysfunction [6].

Although this review primarily focuses on Parkinson’s disease, mitochondrial dysfunction is now widely considered a convergent pathogenic feature across several major neurodegenerative disorders, including Alzheimer’s disease, amyotrophic lateral sclerosis, and Huntington’s disease, in which impairments in oxidative phosphorylation, mitochondrial dynamics, and quality control systems have been extensively documented [7,8]. Nevertheless, the nature and extent of mitochondrial abnormalities vary considerably among these diseases. Parkinson’s disease is mainly associated with complex I impairment and defective mitochondrial quality control mechanisms [9], whereas Alzheimer’s disease is characterized by mitochondrial fragmentation together with progressive bioenergetic decline driven by amyloid-β and tau pathology [10]. In amyotrophic lateral sclerosis, disruption of axonal mitochondrial transport leads to impaired energy supply in long motor neurons, contributing to progressive neuromuscular degeneration [11]. In Huntington’s disease, the mutant Huntingtin protein perturbs mitochondrial dynamics, calcium handling, and bioenergetic stability, thereby increasing neuronal vulnerability, particularly in striatal and cortical regions [12]. These disease-specific mitochondrial alterations ultimately contribute to distinct but overlapping downstream pathological consequences, including synaptic dysfunction, neuronal loss, and progressive neurodegeneration [13].

As summarized in Table 1, mitochondrial dysfunction has been repeatedly observed in major neurodegenerative diseases, including Parkinson’s disease, Alzheimer’s disease, amyotrophic lateral sclerosis, and Huntington’s disease, where disease-specific alterations in mitochondrial bioenergetics, dynamics, and quality control pathways have been reported. However, these findings should be interpreted with caution, as they arise from heterogeneous experimental models and varying disease stages. Accordingly, Table 1 presents a comparative overview of mitochondrial alterations across these disorders, rather than suggesting a unified mechanistic framework.

In Parkinson’s disease, mitochondrial quality control impairment, enhanced mtROS production, and mtDNA-associated neuroinflammatory signaling are particularly prominent features driving disease progression. The role of mitochondrial failure in PD is examined in this review, along with dopaminergic neurodegeneration, mitochondrial quality control, and new treatment approaches.

## 2. From MPTP Toxicity to Complex I-Driven Bioenergetic Failure in Parkinson’s Disease

The association between mitochondrial dysfunction and PD was first recognized in the early 1980s following accidental exposure of drug users to 1-methyl-4-phenyl-1,2,3,6-tetrahydropyridine (MPTP), which induced irreversible Parkinsonian symptoms [14]. Subsequent studies demonstrated that MPTP is metabolized into MPP^+^, a neurotoxic compound selectively taken up by dopaminergic neurons through the dopamine transporter. MPP^+^ inhibits mitochondrial complex I activity and subsequently causes degeneration of dopaminergic neurons in the substantia nigra [15,16].

Based on these findings, further biochemical and pathological studies revealed that complex I deficiency is also present in the substantia nigra of patients with sporadic PD, suggesting that impaired mitochondrial respiration is a shared feature of both toxin-induced and idiopathic forms of the disease [17,18,19]. More recent studies have further linked complex I impairment to broader mitochondrial dysfunction, including excessive reactive oxygen species production and reduced ATP generation, ultimately contributing to bioenergetic deficits in dopaminergic neurons [20,21,22].

Such impairments are harmful to cellular resilience because dopaminergic neurons have high energy requirements. However, there is ongoing debate regarding whether complex I dysfunction is a main initiating event or a later result of upstream pathogenic processes.

## 3. Hierarchical Mitochondrial Quality Control in Parkinson’s Disease

A major question in current studies of mitochondrial dysfunction in PD is whether complex I impairment represents an initiating pathogenic event or develops secondary to prolonged cellular stress [23]. Although toxin-based models initially identified complex I inhibition as a major contributor to neurodegeneration, accumulating evidence from patient-derived tissues and experimental models suggests that complex I dysfunction is part of a broader and progressive process of mitochondrial dysfunction rather than an isolated pathogenic trigger [24,25].

Across different PD models, impaired mitochondria gradually accumulate and exhibit oxidative damage to mitochondrial lipids, proteins, and DNA, accompanied by loss of mitochondrial membrane potential and disruption of mitochondrial integrity [26,27,28,29]. These observations have shifted attention toward the cellular mechanisms responsible for maintaining mitochondrial homeostasis. Mitochondrial quality control (MQC) is now recognized as a key regulatory system that limits the accumulation of damaged mitochondria and preserves mitochondrial function [25]. Importantly, MQC consists of several interconnected processes that act coordinately to maintain mitochondrial integrity under stress conditions [30].

In the early stages of mitochondrial damage, mitochondrial proteostasis serves as the first line of defense. By eliminating oxidized or misfolded proteins, particularly those found in respiratory chain components, proteases such as LONP1 and the ClpXP complex assist maintenance of local mitochondrial function and reduce the accumulation of damaged proteins [31,32]. When mitochondrial damage exceeds the capacity of these proteostatic mechanisms, structural alterations and functional decline become more pronounced.

Another crucial aspect of MQC is mitochondrial dynamics, which includes fusion and fission. While MFN1/2- and OPA1-mediated fusion allows for the interchange of mitochondrial contents and partial functional repair, DRP1-mediated fission encourages the segregation of injured mitochondrial regions. Through continuous remodeling, mitochondrial dynamics help maintain mitochondrial homeostasis and delay the removal of damaged organelles [33,34]. However, in circumstances of prolonged or severe stress, which initiates mitophagy, these adaptive mechanisms are inadequate. By removing highly damaged mitochondria, the PINK1–Parkin pathway prevents additional cellular damage and lessens the build-up of malfunctioning organelles [35,36].

**Figure 1:**
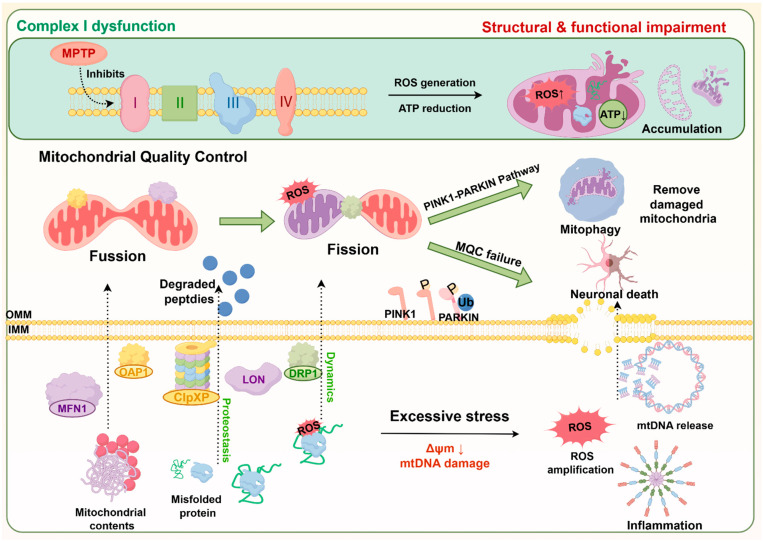
A hierarchical model of mitochondrial quality control in response to mitochondrial dysfunction in Parkinson’s disease.

Even though MQC is increasingly recognized as an interconnected process, many previous studies have focused on particular components such as proteostasis, mitochondrial dynamics, or mitophagy rather than their interactions throughout disease development. Furthermore, the relationship between MQC dysfunction and the progression of PD is still unknown. Variations between experimental models and stage-dependent alterations in MQC further emphasize the need to understand how MQC fluctuates during multiple stages of the disease. The major pathways involved in mitochondrial quality control and mitochondrial dysfunction in PD are illustrated in Figure 1.

Mitochondrial quality control (MQC) operates as a multi-layered system that buffers progressive mitochondrial damage in Parkinson’s disease. Early-stage proteostasis removes damaged proteins (e.g., LONP1 and ClpXP), followed by mitochondrial dynamics (fusion: MFN1/2 and OPA1; fission: DRP1) that redistribute and segregate damage. Under prolonged stress, severely damaged mitochondria are eliminated by mitophagy, mainly through the PINK1–Parkin pathway.

## 4. Mitochondrial Dysfunction-Centered Pathogenic Cascade in Parkinson’s Disease

### 4.1. Mitochondrial ROS as a Central Driver of Redox-Mediated Neurotoxicity

Mitochondrial reactive oxygen species (mtROS) have long been regarded as an initiating factor in the pathogenesis of PD. However, a central paradox persists within this framework: if mtROS were indeed the principal driver of disease progression, why have antioxidant-based therapies consistently failed to provide disease-modifying effects in clinical trials [37]? These findings suggest that oxidative stress alone may not fully explain disease progression in PD.

Recent research suggests that increased mtROS generation may be a result of mitochondrial dysfunction rather than the primary starting event. In particular, MQC deficiency promotes the accumulation of damaged mitochondria, which causes mtROS levels to continuously rise [38,39]. This perspective holds that mtROS is mostly an amplifier of ongoing cellular damage rather than an isolated upstream trigger.

It is necessary to move past the conventional understanding of oxidative stress as a general and discriminating source of damage in order to explain this transformation mechanistically [40]. Current studies in redox biology further indicate that the biological effects of reactive oxygen species are highly dependent on their spatial distribution and local microenvironment because of their short half-life and strong chemical reactivity [41,42]. In dopaminergic neurons, mtROS primarily acts near damaged mitochondria instead of inducing generalized oxidative injury throughout the cell. Under these conditions, mtROS preferentially oxidizes vulnerable mitochondrial components, including cardiolipin and mitochondrial DNA (mtDNA) [43,44]. Progressive oxidative injury disrupts respiratory chain activity, increases electron leakage, and further enhances mtROS production, thereby forming a self-perpetuating cycle of mitochondrial dysfunction [45]. Thus, localized mitochondrial dysfunction becomes a self-sustaining pathogenic cycle as mtROS intensifies the original MQC shortage.

In dopaminergic neurons, mtROS-mediated injury mainly affects two closely related pathogenic pathways: abnormal dopamine metabolism and α-synuclein aggregation associated with mitochondrial membrane alterations.

First, mtROS influences dopamine metabolism at the cytosolic–mitochondrial interface, an area considered particularly vulnerable in nigral neurons. mtROS escaping from damaged mitochondria promotes dopamine auto-oxidation and the formation of reactive dopamine quinones (DAQs) under oxidative conditions [46]. These reactive intermediates have the ability to alter certain lysosomal-related proteins, such as glucocerebrosidase (GCase) [47,48]. As a consequence, lysosomal enzymatic activity and lipid degradation become impaired, linking mitochondrial dysfunction to defects in lysosomal homeostasis [49]. Through this mechanism, mitochondrial oxidative stress contributes to broader disturbances in cellular proteostasis and degradative pathways.

Second, mtROS also promotes interactions between α-synuclein and mitochondrial membranes through oxidative modification of cardiolipin. Cardiolipin is a mitochondria-specific phospholipid enriched in the inner mitochondrial membrane and is highly susceptible to oxidative damage because of its close association with the electron transport chain. Sustained mtROS production induces cardiolipin peroxidation and promotes its translocation from the inner to the outer mitochondrial membrane [50,51]. Externalized cardiolipin enhances α-synuclein binding to mitochondrial membranes and facilitates its aggregation in a lipid-dependent manner [52]. In addition, pathogenic α-synuclein species can impair mitochondrial protein import machinery [53].

Accumulating evidence further suggests that α-synuclein oligomers located at the outer mitochondrial membrane interact directly with the TOM20 translocase complex, thereby disrupting the import of nuclear-encoded mitochondrial proteins [53]. Impaired protein import reduces the replenishment of respiratory chain components, aggravates mitochondrial dysfunction, and further increases mtROS production through enhanced electron leakage [54]. The significance of disturbed mitochondrial proteostasis in the development of PD is further highlighted by recent research showing that mitochondrial protein import stress itself may contribute to neurodegeneration irrespective of overt bioenergetic failure [55].

**Figure 2:**
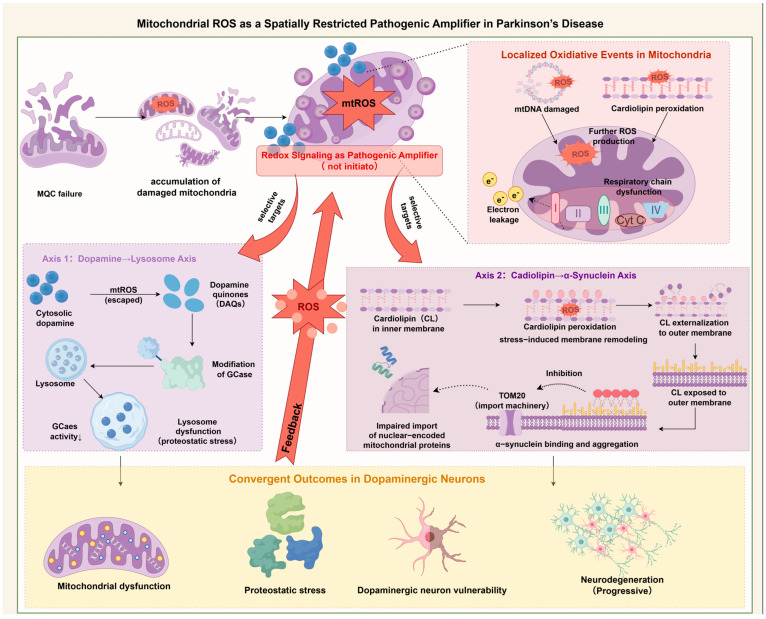
Mitochondrial ROS as a Spatially Restricted Pathogenic Amplifier in Parkinson’s Disease.

All of the available data point to mtROS primarily acting to exacerbate pre-existing mitochondrial damage in susceptible dopaminergic neurons. Instead of producing widespread oxidative damage, mtROS seems to be involved in specific molecular processes linked to cardiolipin remodeling, dopamine oxidation, and α-synuclein aggregation, which gradually impair cellular and mitochondrial function. The limited clinical efficacy of traditional antioxidant medications, which frequently fall short of effectively targeting the particular pathogenic mechanisms implicated in Parkinson’s disease progression, may be partially explained by this mechanism. The main routes linked to mtROS-mediated pathogenic amplification in Parkinson’s disease are compiled in Figure 2.

Damaged mitochondria gradually build up and contribute to the continuous production of mtROS after disruption of MQC. mtROS mostly worsens pre-existing mitochondrial damage by encouraging localized oxidative alterations linked to two main pathogenic mechanisms. Dopamine oxidation and lysosomal dysfunction are involved in one, while cardiolipin remodeling, α-synuclein aggregation, and defective mitochondrial protein import are involved in the other. When combined, these mechanisms worsen mitochondrial dysfunction and make dopaminergic neurons more susceptible to neurodegeneration.

### 4.2. Neuroinflammatory Amplification of Mitochondrial-Derived Danger Signals

Building on the mtROS–lysosome axis previously discussed, current evidence indicates that passive leakage of cellular contents is unlikely to be the cause of the shift from cell-autonomous organelle stress to non-cell-autonomous neuroinflammatory signaling. Rather, it most likely represents a controlled, multi-phase mechanism that connects innate immune activation to intracellular organelle failure. At this stage, damaged mitochondria become an important source of mitochondrial damage-associated molecular patterns (mtDAMPs), linking mitochondrial stress with inflammatory signaling. Current evidence does not support a simple linear progression model. Instead, mitochondrial quality control pathways may initially compensate for organelle damage, whereas persistent cellular stress gradually weakens these protective mechanisms and promotes inflammatory activation [56,57]. However, whether this apparent transition reflects a discrete threshold or a gradual loss of buffering capacity remains unresolved.

Under sustained stress conditions, lysosomal dysfunction and mitochondrial outer membrane permeabilization (MOMP) have both been implicated in the release of mitochondrial contents, particularly mitochondrial DNA (mtDNA). BAX/BAK-dependent pore development within the mitochondrial membrane has been mechanistically connected to mtDNA release during MOMP [58]. Lysosomal membrane permeabilization (LMP) may also reduce lysosomal degradative capability, which would prolong cytosolic exposure to mtDAMPs and decrease the clearance of injured mitochondria. Concurrently, oligomerization of voltage-dependent anion channel 1 (VDAC1) may expedite the release of mitochondrial content and increase the permeability of the mitochondrial membrane [59]. These pathways may work together to inhibit intracellular clearance systems and encourage mtDNA leaking. However, depending on the cellular setting, level of stress, and stage of the disease, these processes’ respective contributions and temporal order probably differ [60]. Whether these mechanisms operate sequentially or in parallel therefore remains an open mechanistic question.

After being released into the cytosol or extracellular environment, mtDNA functions as a strong endogenous danger signal due to its bacterial origin and hypomethylated CpG motifs [61]. Significant evidence indicates that mtDNA activates innate immune sensors, such as the cGAS–STING pathway and Toll-like receptor 9 (TLR9), linking pro-inflammatory signaling channels and type I interferon to mitochondrial damage [59,62]. Although oxidative modification enhances the immunostimulatory activity of mtDNA in several experimental systems, non-oxidized mtDNA is also sufficient to activate these pathways, suggesting that oxidation primarily amplifies rather than initiates immune signaling [63,64,65]. These results are consistent with a hierarchical paradigm where oxidative stress and cellular context regulate signal amplitude and durability, whereas mtDNA release serves as the main initiating signal [66].

Following neuronal mtDNA release and activation of cGAS–STING and TLR9 pathways, inflammatory signaling may be further amplified in microglia through uptake of extracellular mitochondrial debris and interaction with additional pathogenic stimuli such as α-synuclein aggregates [67,68]. This additional amplification reinforces NF-κB-dependent inflammatory primed and inflammasome activation by establishing microglia as a major center for synthesizing mitochondrial and proteinopathy-derived stimuli [69].

At the same time, immunometabolic reprogramming appears to play an important role in sustaining microglial inflammatory activity. Under conditions of mitochondrial dysfunction and HIF-1α stabilization, microglia shift from oxidative phosphorylation toward glycolytic metabolism, thereby supporting the energetic and biosynthetic demands required for persistent cytokine production [70,71]. Although this metabolic transition is commonly viewed as a downstream consequence of immune activation, emerging evidence suggests that altered metabolism may itself further reinforce inflammatory signaling [72].

Sustained microglial activation subsequently propagates inflammatory signaling to neighboring glial populations [73,74]. Pro-inflammatory mediators and reactive nitrogen species released by activated glia impair mitochondrial electron transport chain activity, particularly complex I function, thereby enhancing mitochondrial ROS production and promoting further mtDAMP release [75].

**Figure 3:**
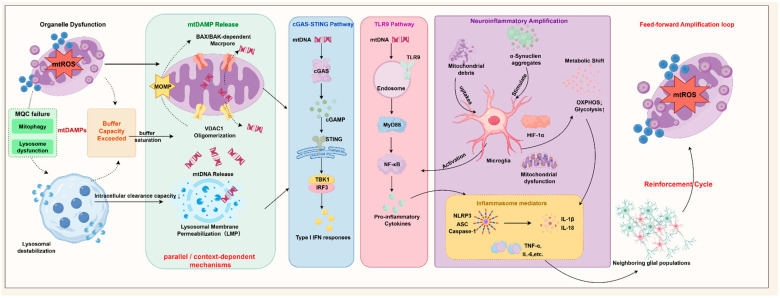
Organelle Dysfunction-Driven mtDAMP Signaling and Microglial Integration in a Feed-Forward Neuroinflammatory Circuit.

All of the available evidence points to the promotion of mtDNA-dependent innate immunological activation by disruption of the lysosome–mitochondria axis, which may be further amplified by microglial activation and modified immunometabolic signaling. These pathways probably entail reciprocal interactions between chronic neuroinflammatory signaling, mitochondrial dysfunction, and reduced intracellular clearance rather than a strictly linear process. The suggested mechanisms connecting mtDNA release to persistent neuroinflammatory responses in Parkinson’s disease are summarized in Figure 3.

Organelle dysfunction leads to mtDNA release via MOMP, VDAC1 oligomerization, and lysosomal permeabilization, activating cGAS–STING and TLR9 pathways. Microglia integrate mitochondrial and protein-derived signals to amplify inflammation through inflammasome activation and metabolic reprogramming, forming a feed-forward neuroinflammatory loop.

### 4.3. PINK1/Parkin-Mediated Mitophagy: A Nexus for Quality Control That Connects Inflammation and Genetics

Mutations in PINK1 (PARK6) and Parkin (PRKN/PARK2) represent some of the most well-established genetic causes of early-onset familial PD, highlighting the critical role of these proteins in MQC [76,77]. Within the broader context of mtDNA-associated neuroinflammatory signaling, the PINK1–Parkin pathway plays an important role in maintaining mitochondrial integrity and limiting the accumulation of mtDAMPs [78,79]. Under conditions of mitochondrial injury, PINK1 accumulates on the outer mitochondrial membrane, where it phosphorylates both ubiquitin and Parkin. This process activates the E3 ubiquitin ligase activity of Parkin, promotes ubiquitination of OMM proteins, and recruits autophagic adaptors to initiate mitophagy [80,81,82]. By removing damaged mitochondria, this procedure prevents their dangerous accumulation and excessive generation of reactive oxygen species.

In familial PD, disruption of either Parkin or PINK1 reduces mitophagy, increases oxidative stress, and leads to dopaminergic neurodegeneration. Deficient PINK1/Parkin signaling may promote innate immune activation by facilitating the storage and release of mitochondrial DNA (mtDNA) and other mtDAMPs in addition to deficient mitochondrial clearance. In addition to its function in mitophagy, the PINK1–Parkin pathway regulates mitochondrial dynamics, including fission and fusion, which further supports the preservation of mitochondrial homeostasis [83].

The canonical PINK1–Parkin signaling cascade has been extensively characterized in genetic models of PD and summarized in recent mechanistic studies. However, although PINK1 and Parkin mutations directly contribute to familial PD, sporadic PD is generally considered to arise through a more complex interaction among aging, environmental exposure, and multiple genetic risk factors [84]. Therefore, dysfunction of the PINK1–Parkin pathway in sporadic PD is more likely to represent a secondary or modulatory event rather than an initiating pathogenic mechanism.

As such, dysfunction of the PINK1–Parkin pathway in sporadic PD is generally considered secondary or modulatory rather than a primary initiating event. This pathway is further modulated by crosstalk with other genes linked to PD. For example, DJ-1 has been found to partially compensate for PINK1 loss in cellular models through redox-dependent processes [85,86], but in transgenic animal models, harmful LRRK2 mutations worsen mitochondrial dysfunction and inhibit PINK1–Parkin signaling [87]. Furthermore, elevated α-synuclein lowers mitophagic flux in primary brain systems, linking protein aggregation to compromised MQC in sporadic PD settings [88].

The functional relevance of the PINK1/Parkin pathway is also shown by results from various experimental models; however, interpretation should account for model-specific differences. Mitochondrial defects primarily occur within neuronal cell bodies rather than axons in Drosophila models missing Parkin, indicating differential vulnerability across neuronal compartments [89]. Similarly, dopaminergic neurons generated from human induced pluripotent stem cells (iPSCs) with PRKN mutations show reduced MQC and heightened vulnerability to stress-induced damage [90]. PINK1 overexpression and pharmacological activation of this pathway both enhance mitochondrial function and lessen neurodegeneration in PD paradigms caused by toxins and genetics in animal models [91,92]. Additionally, it has been demonstrated that pharmacological inhibition of USP30 increases PINK1/Parkin-dependent mitophagy in mammalian cell cultures, indicating the pathway’s possible therapeutic importance [93].

**Figure 4:**
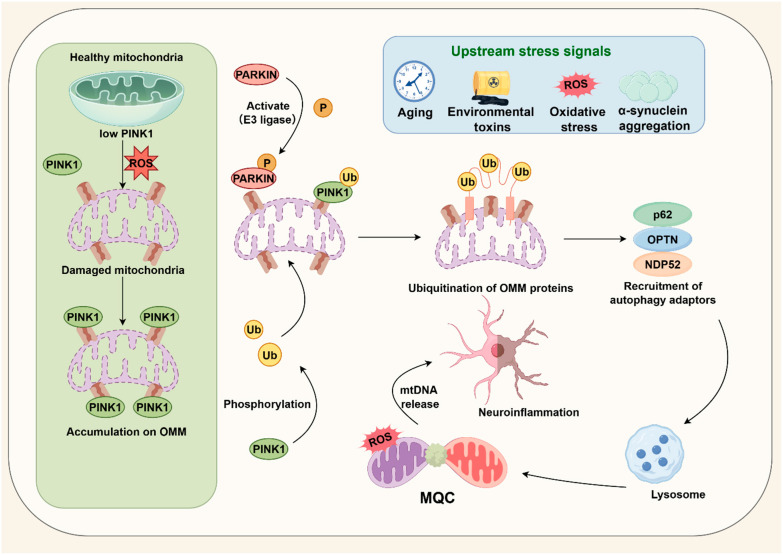
Parkinson’s disease-related deregulation of the PINK1/Parkin mitophagy cascade.

This route may help control innate immune activation linked to mitochondrial failure by encouraging the removal of damaged mitochondria and restricting the release of mtDNA. While disruption of PINK1/Parkin signaling offers a strong biological basis for mitochondrial dysfunction in familial PD, its effects in sporadic PD are probably more complicated and may entail interactions with oxidative stress, protein aggregation, and other pathogenic pathways. The interactions between the PINK1–Parkin pathway and other stress-related pathways linked to the development of Parkinson’s disease are summarized in Figure 4.

PINK1 accumulates on the OMM as a result of mitochondrial injury. PINK1 then attracts, phosphorylates, and activates the E3 ligase Parkin, which causes OMM proteins to be ubiquitinated. In order to ensure MQC, this polyubiquitin chain makes it easier for autophagy adaptors (including p62, OPTN, and NDP52) to be recruited and convey damaged mitochondria to lysosomes for destruction. Upstream stress signals, such as aging, environmental pollutants, ROS, and α-synuclein aggregation, which are widely linked to sporadic PD, might directly affect this protective system. The accumulation of faulty mitochondria and gradual neurodegeneration are ultimately caused by this pathway’s failure.

### 4.4. An Epitranscriptomic Controller of Mitochondrial Homeostasis by RNA Modification

In addition to genetic mechanisms such as the PINK1/Parkin pathway, epigenetic and epitranscriptomic regulation have increasingly been implicated in mitochondrial dysfunction and neuroinflammatory susceptibility in PD, particularly in sporadic forms of the disease. Among these regulatory mechanisms, N^6^-methyladenosine (m^6^A), the most abundant internal modification in eukaryotic mRNA, has emerged as a potential contributor to mitochondrial homeostasis. m^6^A modification is primarily mediated by the METTL3/METTL14 methyltransferase complex and influences the expression of genes involved in oxidative phosphorylation, mitochondrial function, and cellular stress responses [94,95].

Accumulating evidence from PD models suggests that dysregulation of m^6^A modification is associated with impaired mitochondrial respiration and increased oxidative stress in dopaminergic neurons [94,96]. Crucially, whereas abnormal METTL3 activity increases dopaminergic neuronal susceptibility under neurotoxic circumstances, restoring m^6^A levels through METTL14 enhances mitochondrial respiratory capacity, stabilizes membrane potential, and decreases ROS generation [96,97,98]. Taken together, these findings suggest that m^6^A serves as a modulatory layer that influences mitochondrial quality control capacity and stress adaptation rather than as a primary pathogenic driver.

**Figure 5:**
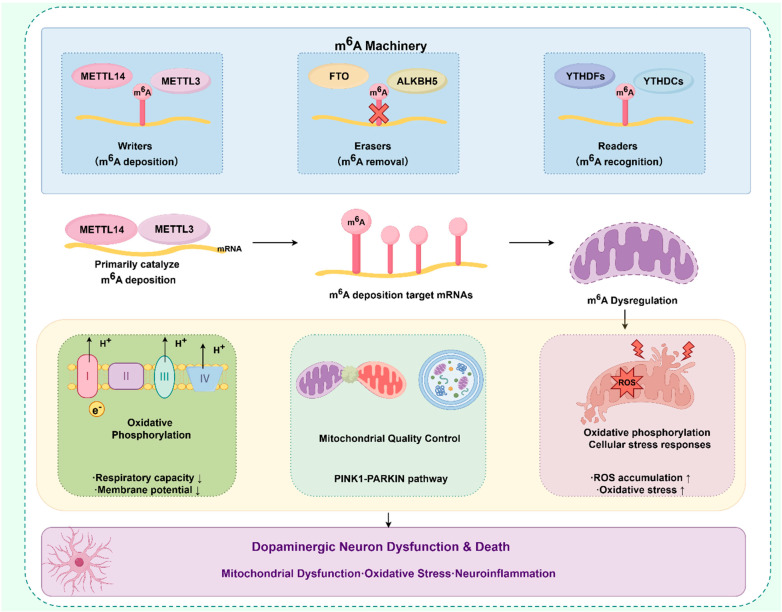
m^6^A-mediated control of mitochondrial activity in Parkinson’s disease.

Although research in this area remains at an early stage, current evidence supports a broader role for RNA methylation in regulating neuronal responses to mitochondrial stress. At the same time, differences among experimental systems indicate that additional functional studies and transcript-specific analyses are still needed to clarify the precise contribution of m^6^A modification to PD pathogenesis [94,97]. Figure 5 summarizes the proposed relationship between m^6^A-mediated regulation, mitochondrial homeostasis, and stress-related neuronal dysfunction in PD.

The “writers” (METTL3/METTL14 complex), “erasers” (FTO and ALKBH5), and “readers” (e.g., YTH domain-containing proteins) that make up an RNA modification machinery dynamically control m^6^A on target mRNAs, which undergo deposition, removal, and recognition. m^6^A change affects oxidative phosphorylation, mitochondrial quality control, and cellular stress responses by altering the expression of genes related to mitochondrial function. m^6^A dysregulation has been linked to increased buildup of ROS, reduced membrane potential, and compromised mitochondrial respiration. Additionally, new data points to a possible involvement of m^6^A in PINK1-Parkin-mediated mitophagy and other mitochondrial quality control processes. Together, these changes lead to dopaminergic neuronal damage and mitochondrial dysfunction in PD.

## 5. Tackling Mitochondrial-Centered Pathogenic Loops in Parkinson’s Disease Has Therapeutic Implications

Based on the pathogenic mechanisms discussed above, current therapeutic strategies targeting mitochondrial dysfunction in PD can generally be divided into three major categories: mitochondrial protection, enhancement of mitophagy and MQC, and modulation of neuroinflammatory signaling. Rather than focusing on a single downstream mechanism, these approaches increasingly aim to simultaneously regulate mitochondrial stress, impaired mitochondrial clearance, and chronic inflammatory activation.

Although their therapeutic usefulness is still restricted, small compounds like MitoQ, Coenzyme Q10, and SkQ1 have been thoroughly studied for their capacity to lower mitochondrial oxidative burden on the level of mitochondrial protection [37,99,100]. In parallel, strategies aimed at increasing intracellular NAD^+^ levels, such as nicotinamide mononucleotide (NMN) and nicotinamide riboside (NR), have been explored for their potential to enhance mitochondrial bioenergetics and improve cellular stress resistance [101,102].

A second therapeutic direction focuses on restoring mitochondrial clearance pathways. Compounds such as spermidine and urolithin A have been reported to enhance autophagy and mitophagy activity [103,104]. In addition, pharmacological modulation of the PINK1–Parkin pathway and its upstream regulators, including AMPK activators such as metformin and mTOR inhibitors such as rapamycin and related analogs, is being investigated as a potential strategy to improve MQC function [105,106,107].

Another emerging strategy involves suppression of mitochondrial dysfunction-associated inflammatory signaling. Experimental inhibitors targeting the NLRP3 inflammasome, including MCC950, as well as modulators of the cGAS–STING pathway such as the small-molecule STING inhibitor H-151, are currently being investigated for their potential to reduce chronic neuroinflammatory responses associated with mitochondrial injury [108,109,110].

Despite these advances, no single therapeutic approach has yet demonstrated robust disease-modifying efficacy in PD. This limitation likely reflects the complex and multifactorial nature of PD pathogenesis, including pathway redundancy and stage-dependent cellular vulnerability [111]. As a result, combination strategies that can target mitochondrial stress, impaired mitochondrial clearance, and inflammatory amplification simultaneously are receiving more attention. Future therapeutic development will probably also depend on better delivery systems with increased cell type specificity and improved blood–brain barrier penetration [112].

As summarized in Table 2, emerging interventions can be broadly categorized into mitochondrial protection, mitophagy enhancement, and suppression of mitochondria-associated inflammatory pathways. These approaches collectively highlight a growing shift from single-target interventions toward integrated therapeutic strategies aimed at simultaneously restoring mitochondrial homeostasis and limiting chronic neuroinflammation.

## 6. Controversies and Challenges in Mitochondrial Research for Parkinson’s Disease

Clinical treatments focusing on mitochondrial pathways, from antioxidants to mitochondrial enhancers, have mainly failed to yield significant disease-modifying effects despite decades of data connecting mitochondrial dysfunction to Parkinson’s disease. This translational gap reflects a fundamental unanswered question in the field: does mitochondrial dysfunction drive neurodegeneration directly or does it emerge as a secondary consequence within larger pathogenic feedback networks involving neuroinflammation, α-synuclein proteotoxicity, and impaired cellular proteostasis?

The significant heterogeneity of PD adds to this uncertainty. While mitochondrial changes may vary significantly between illness stages, neuronal subpopulations, and patient-specific molecular characteristics, familial and sporadic forms of the disease probably involve different pathogenic pathways. However, a lot of experimental paradigms still regard PD as a biologically homogeneous disorder, which may oversimplify the intricate nature of mitochondrial pathology.

Mechanistic interpretation is nevertheless limited by methodological issues. Acute toxin-based models incompletely mimic the chronic and progressive characteristics of PD, whereas human iPSC-derived systems generally lack crucial aging-associated and microenvironmental aspects. In parallel, postmortem omics investigations provide only static snapshots of end-stage pathology and remain insufficient to understand the temporal dynamics of mitochondrial failure during disease progression.

The idea that PD is not a single-pathway pathology and that mitochondrial dysfunction should be viewed as a dynamic network-level process rather than an isolated pathogenic event is supported by these conceptual and technical constraints taken together. Moving away from single-target therapy paradigms toward precision-based, stage-specific interventions driven by reliable biomarkers and integrated molecular profiling will probably be necessary for future advancements. In the end, combination approaches that can concurrently regulate proteotoxicity, poor quality control, mitochondrial stress, and neuroinflammatory signaling may be more successful in reducing the rate of disease development.

## Figures and Tables

**Table 1:** Comparison of mitochondrial dysfunction and associated pathological consequences across major neurodegenerative diseases.

Disease	Mitochondrial Abnormalities	Major Pathogenic Consequences	Representative Evidence
Parkinson’s disease (PD)	Complex I impairment; defective mitochondrial quality control	Dopaminergic neuron loss; α-syn aggregation; neuroinflammation	mtROS; mtDNA signaling
Alzheimer’s disease (AD)	Mitochondrial fragmentation; bioenergetic decline	Aβ accumulation; tau pathology; synaptic dysfunction	Aβ/tau-related mitochondrial dysfunction
Amyotrophic lateral sclerosis (ALS)	Impaired axonal mitochondrial transport	Motor neuron degeneration; neuromuscular dysfunction	Axonal energy failure
Huntington’s disease (HD)	Mitochondrial dynamics disruption; Ca^2+^ dysregulation	Striatal and cortical neurodegeneration	Mutant huntingtin-associated dysfunction

**Table 2:** Therapeutic strategies targeting different stages of mitochondrial dysfunction in Parkinson’s disease.

Pathogenic Process	Representative Targets	Therapeutic Approaches	Expected Outcomes
Oxidative stress amplification	mtROS	MitoQ, CoQ10, SkQ1	Reduction in oxidative damage
Bioenergetic dysfunction	NAD+ metabolism	NMN and NR	Improved mitochondrial respiration
Impaired MQC	PINK1/Parkin; AMPK/mTOR	Spermidine, Urolithin A, Metformin, Rapamycin	Enhanced mitophagy and mitochondrial turnover
mtDNA-mediated inflammation	cGAS-STING; TLR9	H-151 and related inhibitors	Reduced innate immune activation
Inflammasome activation	NLRP3	MCC950	Suppression of neuroinflammation
Multiple pathogenic pathways	Combined therapy	Multi-target strategies	Comprehensive disease modification

## Data Availability

Data sharing is not applicable to this article as no new datasets were generated or analyzed during the current study. All information presented in this review is based on publicly available published literature.
